# Post-transfusion purpura in an African-American man due to human platelet antigen-5b alloantibody: a case report

**DOI:** 10.1186/1752-1947-6-420

**Published:** 2012-12-12

**Authors:** Filipa Lynce, Fang Yin, Kirsten Alcorn, Vera Malkovska

**Affiliations:** 1 Hematology/Oncology, Washington Cancer Institute, Medstar Washington Hospital Center, 110 Irving Street NW C2151, Washington, DC 20010, USA; 2 Hematology/Oncology, Hematology Branch, NHLBI, National Institutes of Health, 10 Center Drive (MSC 1475), Building 10, CRC 4-5140, Bethesda, MD, 20892, USA; 3 Transfusion Services, Medstar Washington Hospital Center, 110 Irving Street NW G012, Washington, DC, 20010, USA

## Abstract

**Introduction:**

Post-transfusion purpura is a rare immunohematological disorder characterized by severe thrombocytopenia following transfusion of blood components and induced by an alloantibody against a donor platelet antigen. It occurs primarily in women sensitized by pregnancy and is most commonly caused by anti-human platelet antigen-1a antibodies. Here, we describe what we believe to be the first documented case of an African-American man who developed post-transfusion purpura due to an anti-human platelet antigen-5b alloantibody after receiving multiple blood products.

**Case presentation:**

A 68-year-old African-American man initially admitted with atrial flutter was started on anticoagulation treatment, which was complicated by severe hematemesis. On days 4 and 5 of hospitalization, he received six units of packed red blood cells, and on days 4, 13 and 14 he received plasma. His platelet count began to drop on day 25 and on day 32 reached a nadir of 7 × 10^9^/L. His platelet count increased after receiving intravenous immune globulin. An antibody with reactivity to human platelet antigen-5b was detected by a solid-phase enzyme-linked immunoassay. Our patient was homozygous for human platelet antigen-5a.

**Conclusion:**

This case emphasizes the importance of including post-transfusion purpura in the differential diagnosis for both men and women with acute onset of thrombocytopenia following transfusion of blood products. The prompt recognition of this entity is crucial for initiation of the appropriate management.

## Introduction

Post-transfusion purpura (PTP) is a rare immunohematological disorder, characterized by a potentially life-threatening thrombocytopenia induced in a transfusion recipient by an alloantibody against a donor platelet antigen, most commonly human platelet antigen (HPA)-1a [1,2]. Most frequently it follows transfusion of cellular blood components but it has also been described after transfusions of plasma [3]. The diagnosis is confirmed by detecting a circulating alloantibody to a common platelet antigen, and by demonstrating that the patient’s own platelets lack this antigen. PTP associated with anti-HPA-5b antibody has been rarely reported [4] and appears to have unique characteristics. Here, we describe what we believe to be the first documented case of an African-American man who developed PTP due to an anti-HPA-5b alloantibody after receiving multiple blood products.

## Case presentation

A 68-year-old African-American man was initially admitted with atrial flutter and was started on anticoagulation treatment, which was complicated by hematemesis requiring transfer to our intensive care unit (ICU) on day 4 of hospitalization. He had a past medical history of packed red blood cell (PRBC) transfusion in 2002. On admission, his blood count showed hemoglobin 13.6g/dL, red blood cell count 5.14 × 10^12^/L, and platelets 265 × 10^9^/L. His prothrombin time was 17.6 seconds (normal range: 11.8 to 14.5 seconds), activated partial thromboplastin time was 30.4 seconds (normal range: 23.0 to 35.0 seconds) and international normalized ratio was 1.5. His ICU course was further complicated by *Moraxella catarrhalis* pneumonia, for which he completed a course of imipenem, and by *Clostridium difficile* colitis treated with oral vancomycin.

**Figure 1:**
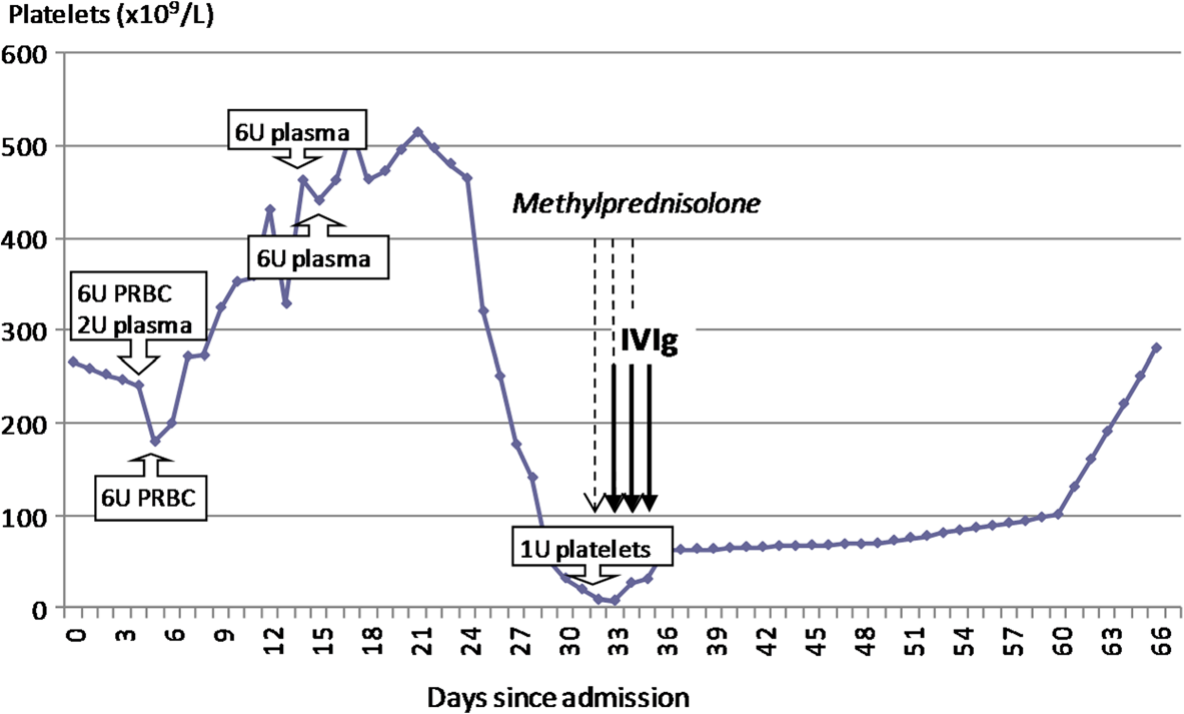
**Time relationship between platelet counts, blood products and medication administered.** U, unit(s); PRBC, packed red blood cell; IVIg, intravenous immune globulin.

While in the ICU, our patient received six units of PRBC and two units of plasma on day 4 of hospitalization, six units of PRBC on day 5 and six units of plasma on day 13 and day 14. He clinically improved and was transferred to the medical ward on day 21. His platelet count began to drop rapidly on day 25 (Figure 1) and on day 29 his platelets decreased to 51 × 10^9^/L and he developed hematuria. He was afebrile and had no signs of infection. On day 30, his platelet count dropped to 31 × 10^9^/L. His prothrombin time and activated partial thromboplastin time were normal. A peripheral blood smear was remarkable for large platelets that were decreased in number. The differential diagnosis at this time included drug-related thrombocytopenia, PTP and immune thrombocytopenic purpura. Several medications were discontinued. However, his platelet count continued to drop, reaching a nadir of 7 × 10^9^/L on day 32. On that same day, our patient received methylprednisolone 1mg/kg and one unit of single-donor platelets. On day 33, intravenous immune globulin (IVIg) was begun at a dose of 700mg/kg daily for three doses. Two days after the first dose of IVIg, his platelet count increased to 46 × 10^9^/L without further transfusions and the hematuria resolved. An enzyme-linked immunosorbent assay for antibodies to platelet surface glycoproteins demonstrated the presence of an antibody with reactivity to HPA-5b. Prior to the administration of IVIg, an antibody with reactivity to HPA-5b was detected by a solid-phase enzyme-linked immunoassay commercial kit (Gen-Probe, Inc., San Diego, CA, USA), cleared for *in vitro* diagnostic use by the US Food and Drug Administration. The absorbance (optical density) of our patient’s sample was 0.27 (negative control: 0.13). Our patient’s platelet genotyping by polymerase chain reaction and fluorescent hydrolysis revealed HPA-5a/5a (Mayo Clinic Laboratories, Rochester, MN, USA). Methylprednisolone was discontinued on day 34 of admission. On day 36, our patient was discharged without bleeding manifestations and with a platelet count of 61 × 10^9^/L. Twenty-two days after discharge, his platelet count had increased to 280 × 10^9^/L.

## Discussion

The only previously published case of PTP induced by the anti-HPA-5b alloantibody was described in a white multiparous woman after an elective hysterectomy [4]. To the best of our knowledge, our case represents the first reported episode of confirmed PTP due to anti-HPA-5b in an African-American man. The anti-HPA-5b alloantibody developed after a total of 26 units of blood products given for acute hemorrhage in the setting of anticoagulation and multiple infections. Our patient’s platelet count dropped after he recovered from his infection, at a time when he was clinically improving. The first drop in platelet count was recorded 20 to 21 days after transfusions of PRBC and 11 to 21 days after plasma transfusion (Figure 1). Both PRBC and plasma transfusions have been associated with PTP [5]. The diagnosis of PTP in our patient was supported by serologic studies demonstrating an alloantibody directed against HPA-5b and by genotyping indicating that our patient was homozygous for HPA-5a.

The incidence of PTP caused by anti-HPA-1a antibody has been reported as one case per 50,000 to 100,000 units of transfused blood components, but this is probably an underestimate [6,7]. PTP caused by anti-HPA-5b appears even rarer. This cannot be explained by the frequency of HPA-5a and HPA-5b antigens in the general population. The majority of the world’s population has genotype 5a/5a, with the highest frequencies of HPA-5b in the United States found among African-Americans at 0.21, while Caucasians have HPA-5b gene frequency of 0.11 [8]. The calculated mismatch probability in the HPA-5 system after random transfusions is higher than in the HPA-1 system [9], and yet the vast majority of reported cases of PTP are associated with the latter. The apparent rarity of HPA-5b-associated PTP could be due to multiple factors including decreased reporting, failure to make the diagnosis or decreased antigenicity of the HPA-5 compared to HPA-1 antigens [10]. The former are expressed at a much lower density and are located on a different platelet glycoprotein, which could result in less antibody formation [3]. Interestingly, anti-HPA-5b antibody is more frequently associated with neonatal alloimmune thrombocytopenia [11]. This may be explained by the different pathogenesis of neonatal alloimmune thrombocytopenia compared with PTP, where the alloantibody causes destruction of not only HPA-5b-positive but also HPA-5b-negative platelets [12].

## Conclusion

Our report highlights the importance of considering PTP in the differential diagnosis for both men and women with acute onset of thrombocytopenia following transfusion of blood products. Antibodies targeting antigens other than HPA-1a, such as HPA-5b, can cause potentially life-threatening bleeding and should be tested for if PTP is suspected.

## Consent

Written informed consent was obtained from the patient for publication of this case report and accompanying images. A copy of the written consent is available for review by the Editor-in-Chief of this journal.

## Competing interests

The authors declare that they have no competing interests.

## Authors’ contributions

FL wrote the manuscript. FY, KA and VM reviewed and edited the manuscript. All authors read and approved the final manuscript.
